# In Vivo Applications of Dendrimers: A Step toward the Future of Nanoparticle-Mediated Therapeutics

**DOI:** 10.3390/pharmaceutics16040439

**Published:** 2024-03-22

**Authors:** Krzysztof Sztandera, José Luis Rodríguez-García, Valentín Ceña

**Affiliations:** 1 Unidad Asociada Neurodeath, Instituto de Nanociencia Molecular, Universidad de Castilla-La Mancha, 02006 Albacete, Spain; krzysztof.sztandera@uclm.es; 2 Centro de Investigación Biomédica en Red en Enfermedades Neurodegenerativas, Instituto de Salud Carlos III, 28029 Madrid, Spain; 3 Department of Internal Medicine, General University Hospital of Albacete, 02006 Albacete, Spain; jlrg61@gmail.com

**Keywords:** dendrimers, cellular uptake, in vivo, drug delivery, gene delivery, theragnostic

## Abstract

Over the last few years, the development of nanotechnology has allowed for the synthesis of many different nanostructures with controlled sizes, shapes, and chemical properties, with dendrimers being the best-characterized of them. In this review, we present a succinct view of the structure and the synthetic procedures used for dendrimer synthesis, as well as the cellular uptake mechanisms used by these nanoparticles to gain access to the cell. In addition, the manuscript reviews the reported in vivo applications of dendrimers as drug carriers for drugs used in the treatment of cancer, neurodegenerative diseases, infections, and ocular diseases. The dendrimer-based formulations that have reached different phases of clinical trials, including safety and pharmacokinetic studies, or as delivery agents for therapeutic compounds are also presented. The continuous development of nanotechnology which makes it possible to produce increasingly sophisticated and complex dendrimers indicates that this fascinating family of nanoparticles has a wide potential in the pharmaceutical industry, especially for applications in drug delivery systems, and that the number of dendrimer-based compounds entering clinical trials will markedly increase during the coming years.

## 1. Introduction

Over the last few years, the development of nanotechnology has significantly increased. Nanotechnology allows researchers to synthesize many different nanostructures with controlled sizes, shapes, and properties. Such nanostructures include, among others, metal nanoparticles [1], polymeric micelles [2], polymersomes [3], dendrimers [4,5], and liposomes [6], with dendrimers being the best-characterized of these to date [7]. The name “dendrimer” comes from the combination of the Greek words “dendros” meaning “tree or branching”, and “meros” meaning “part of”. The high number of chemically modifiable terminal groups in their surface, as well as the void spaces present in their interior makes dendrimers excellent delivery agents for different therapeutic agents, including genetic material and drugs. Initial attempts to synthesize these branched polymers were first made in the 1940s. However, it was not until 1985 that the team of Donald Tomalia first described and synthesized poly(amidoamine) or PAMAM dendrimers [8]. To date, more than 100 families of dendrimers differing in their initiator cores, branching units and terminal moieties have been synthesized and evaluated [9]. Some of them, such as PAMAM [10], poly-L-lysine (PLL), bis-methyl propionic acid [11], and poly(propyleneimine) (PPI) dendrimers [12], have been commercialized while others like phosphorus dendrimers [13], Janus dendrimers [14], peptide dendrimers [15], rotaxane dendrimers [16], and carbosilane dendrimers [17] among others [18,19,20,21,22] have not. Amphiphillic Janus dendrimers and glycodendrimers are water-soluble mimics of biological membranes, including cell membrane glycans that self-assemble into unilamellar or onion-like dendrimersomes with predictable dimensions [23]. This dendrimer family has shown efficacy for the in vivo targeted delivery of mRNA to different organs based on changes in their chemical structure [24].

## 2. Dendrimer Structure

**Figure 1:**
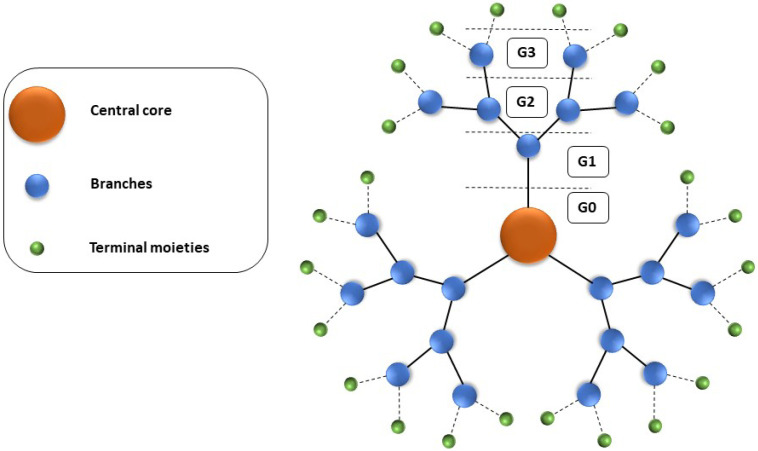
Scheme of the dendrimer structure. For a detailed explanation, see the text.

These interesting nanoparticles are three-dimensional sphere-shaped and monodispersed polymers with symmetrical branches [25]. They consist of three components: the central core, branches, and terminal groups (Figure 1). Branches are attached to the central core and end in terminal moieties that play a role as a dendrimer scaffold. As the number of branches increases, the dendrimer generation grows, and after generation 4 (G4), these nanoparticles adopt a globular structure [10] resembling the natural vesicles found in cells.

The synthesis of dendrimers can be strictly controlled, which provides high-purity homogeneous, monodispersed products with a well-defined structure, size, density, and uniform molecular weight [8,26,27]. This distinguishes them from classical linear polymers, that usually generate polydisperse products with different molecular weights. 

**Figure 2:**
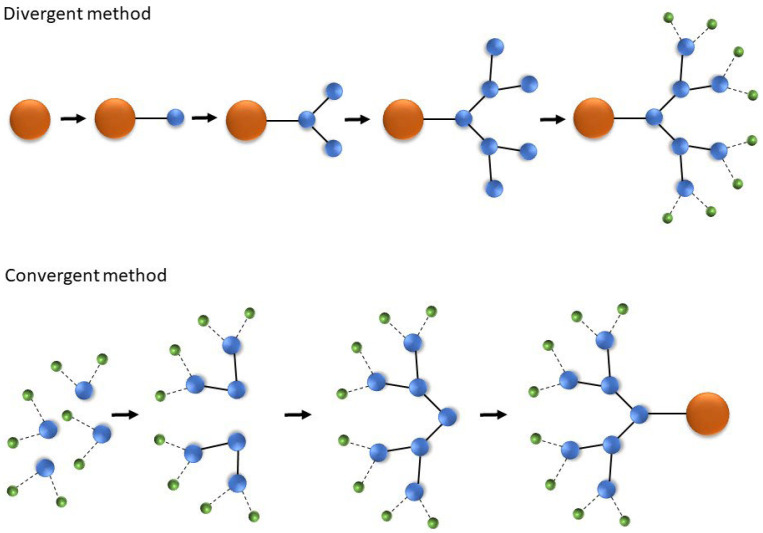
Scheme of the divergent and convergent methods for dendrimer synthesis. Symbols are the same as in Figure 1. For a detailed explanation, see the text.

There are two main ways to synthesize dendrimers: a divergent and a convergent approach (Figure 2). In the first method, monomers are added to the central core to engender the desired dendrimer generation. In contrast, during convergent synthesis, large segments (whole branches) are synthesized and then joined together to generate the dendrimer [26]. The use of click chemistry in the synthesis of dendrimers is also becoming increasingly popular. This system allows for the quick modification of substances without interfering with their structure [28]. Indeed, several reports about click chemistry in dendrimer synthesis with the use of thiol–yne, azide–alkyne, and Diels–Alder reactions have already been published [29,30,31].

Dendrimer synthesis requires a repeated series of reactions that increase dendrimer size and generation. Of note, there is a threshold generation level whereafter the attachment of further elements is impossible because the terminal groups form a closed membrane, with this phenomenon being referred to as the starburst effect [31]. Therefore, the synthesis of G10 is considered the highest possible level [32]. Furthermore, appropriate monomer selection is crucial in the development of dendrimers because the size, solubility, biocompatibility, multivalency, and ability to interact with biological systems depend on these initial units [33,34,35]. Moreover, manipulation of the dendrimer surface can prolong their blood circulation time or change their biodistribution in vivo. 

**Figure 3:**
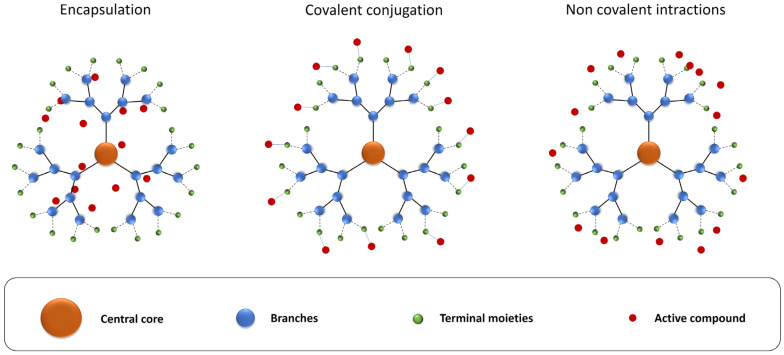
Main strategies for transporting active compounds by dendrimers. For a detailed explanation, see the text.

The terminal groups of dendrimers can also be functionalized with biologically active molecules to provide them with wider application possibilities [36,37], thereby allowing them to encapsulate compounds (Figure 3). These cavities are usually hydrophobic and can generate hydrogen bonds to the transported compounds [38,39,40], which protects drugs from biodegradation before reaching the desired site of action [38]. However, because this premise is based on hydrophobic interactions, only hydrophobic compounds are used in this way. In addition to the use of internal cavities for storage, a wide range of terminal groups can also be conjugated covalently on the surface of dendrimers through specific linkers, including those sensitive to changes in pH or temperature, enzymatic or redox reactions, light irradiation, or hypoxia [41]. Non-covalent interactions between the terminal groups of dendrimers and different compounds do not interfere with the structure of these compounds but could be unstable in vivo. Thus, covalent bonding is the most stable connection between dendrimers and drugs but requires chemical modification of the compound by attaching linkers which could, in turn, change their properties [42] (Figure 3). The perspectives for the use of dendrimer-based systems as nanomedicines [43], biological adhesives [44], imaging agents [45], and in gene therapy [46] are all very promising. Dendrimers can be also used as vectors for the delivery of genes [47], nucleic acids [48], and drugs [49]. Moreover, research is currently underway to use dendrimers as adjuvants in vaccines [50] based on evidence that nanoparticles protect drugs from biodegradation, prolong their half-life in blood, improve their solubility, and provide targeted delivery [51]. 

## 3. Cellular Dendrimer Uptake

**Figure 4:**
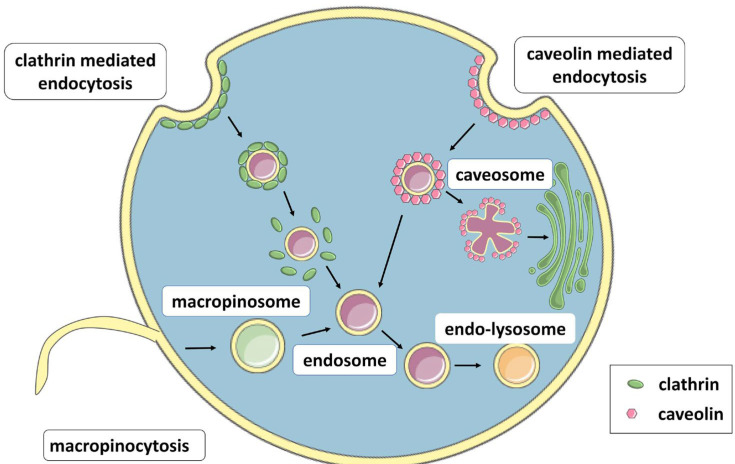
Main pathways for the intracellular uptake of dendrimers. For a detailed explanation, see the text.

The main use of dendrimers to date is in the transport and delivery of different compounds to cell interiors. Thus, intracellular uptake is one of their most studied properties. Two main mechanisms are responsible for the intracellular transport of dendrimers: clathrin-mediated endocytosis (CME) and caveolin-mediated endocytosis (CVME). However, under some specific conditions, macropinocytosis can also be engaged in this process (Figure 4). During CME, clathrin is recruited to specialized regions of the cell membrane where it coats endocytic vesicles measuring 70–150 nm [52]. After internalization by the cell, the vesicles lose the clathrin coat and form early endosomes which later evolve into late endosomes and fuse with lysosomes [53]. This internalization pathway is common for receptor–ligand complexes such as low-density lipoprotein (LDL) particles [54] or epithelial growth factor [55], virus entry [56], and the uptake of neurotransmitters [57]. In turn, CMVE starts by generating 60–80 nm invaginations of the cell membrane in which proteins participating in endocytosis (e.g., caveolin-1) bind to lipid rafts. These vesicles either fuse with other caveolin vesicles to form caveosomes or with early endosomes, which can be transported to the Golgi apparatus or the smooth endoplasmic reticulum [58]. This pathway is used for, among other substances, the internalization of cholesterol [59], TGF-β receptor signaling [60] or toxins [61]. Macropinocytosis starts by creating vacuoles, termed macropinosomes, of variable sizes. After these enter cells, their pH decreases and they fuse with late endosomes or lysosomes [58]. This pathway is mainly used for the uptake of proteins [62], viruses [63], and antigens [64].

The type and efficacy of the cellular uptake of dendrimers is strongly dependent on the cell type, generation route, and surface charge of the nanoparticles in question. For instance, G4 PAMAM dendrimers are better internalized than lower generations [58]. Moreover, epithelial cells have a negative charge on their surface because of the presence of phosphate groups. Combined with a positive charge on the surface of cationic dendrimers, this creates electrostatic interactions that increase the efficiency of endocytosis [65]. Thus, it is extremely important to choose a dendrimer with an appropriate surface charge for every cell type. For example, PAMAM dendrimers with -NH_2_ moieties in MCF-7 cells are internalized by CME and micropinocytosis. However, in the case of A549 cells, they are internalized by both the CME and CVME pathways [66]. Furthermore, the chemical modification of dendrimers is also relevant: for instance, naked G4.5 PAMAM dendrimers are usually internalized through the CME, whereas after PEGylation (the modification of biological molecules by covalent conjugation with polyethylene glycol [PEG]), the main endocytic pathway changes to CVME [67].

The ideal nanocarriers should protect cargo compounds from biodegradation and release them into the targeted area. Moreover, they must also be biocompatible and not cause side effects [67]. Because of their shape, structure, and nanometric size, dendrimers can also penetrate into tumoral environments and be retained in the interstitium of tumors, a property termed the enhanced permeability and retention effect [51]. In addition, the possibility of almost unlimited dendrimer modifications by, for example, conjugation with antibodies, carbohydrates, folic acid (FA), or PEG, or by attaching active components through encapsulation, conjugation, or noncovalent interactions, makes these nanoparticles a promising alternative to regular cancer treatments [51]. In fact, nanosystems comprising dendrimers and drugs that result in improved cellular uptake and increased retention time compared to free drug delivery systems have been widely described in the academic literature [68,69,70].

## 4. In Vivo Use of Dendrimers

The cytotoxic activity, hemolytic properties, cellular uptake, or ability to transport and release different cargo compounds of many dendrimers have already been studied in vitro [9,71]. However, studies in isolated groups of cells do not accurately model the difficulties nanoparticles must overcome to reach their targets in vivo. These include biological barriers such as the blood–brain barrier (BBB), short plasma circulation times, hepatic or renal clearance, and limited distribution to sites of drug action [72]. Hence, more complex in vivo models of disease are now shedding new light on the usability of dendrimers in living systems. Studies performed at this stage aim to evaluate acute toxicity, dose dependence, and long-term toxicity in animal models, with parameters including pharmacodynamics and pharmacokinetics also being important.

### 4.1. Dendrimers as Carriers for Active Compounds

**Figure 5:**
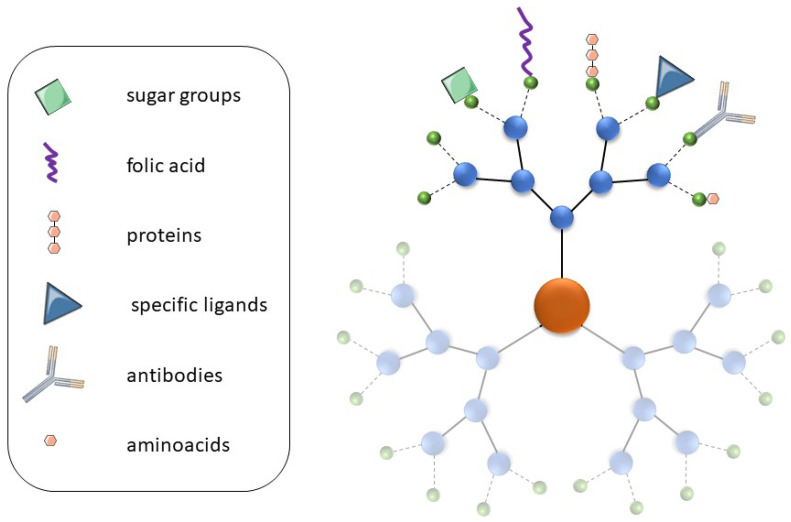
The targeting methods of dendrimers. For a detailed explanation, see the text.

There is intense interest in dendrimers as delivery vehicles, especially for anti-cancer, anti-neurodegenerative, and anti-inflammation drugs [73,74,75,76], with the most important role of dendrimers in these systems being the efficient transport of drugs to the site of action. The almost infinite possibilities for dendrimer functionalization mean that compounds such as antibodies [77,78], folic acid [79], amino acids [80,81], sugar groups [82,83], or other specific ligands can be attached to their surface so that these nanosystems can target specific cells and tissues (Figure 5) [84]. For instance, these dendrimers could be designed to target cells with specific receptors or other substances secreted specifically by a given tumor type. Indeed, such modifications might also enable these molecules to cross the BBB [85]. Moreover, the use of pH-sensitive linkers can be used to release the drugs they carry only in a specific environment [86]. Thus, all the above-mentioned strategies could be combined to help improve drug efficiency and decrease side effects.

To date, most in vivo studies have focused on PAMAM dendrimers, although a few other dendrimer types have also been evaluated. For example, Chen et al. used phosphorus dendrimers to prepare micelles in order to encapsulate doxorubicin (DOX). They showed that this nanosystem can decrease the size of breast cancer tumor xenografts in mice by upregulating Bax, PTEN, and p53 [87]. Another group also used DOX combined with peptide dendrimers to target pancreatic ductal adenocarcinoma xenografts in mice, resulting in increased accumulation and internalization of the drug into these tumors. Additionally, these authors found that the efficacy of DOX was greater when it was co-administered with the dendrimer [88]. Furthermore, they reported that in a zebrafish model, the release of DOX in response to γ-radiation was increased when delivered by PAMAM dendrimers modified with L-cysteine because the latter acts as a radiosensitizer that allows for the release of the drug and inhibits cancer growth [89].

The use of PAMAM dendrimers to deliver other anti-cancer agents has also been extensively studied. For example, Bhadra et al. exploited naked and PEGylated PAMAM dendrimers to transport 5-fluorouracil and verified that this nanosystem was stable and biocompatible in albino rats by measuring the serum levels of the drug. Thus, this nanosystem proved to be safe for animals; and moreover, PEGylation reduced drug leakage and hemolysis, thereby indicating that PAMAM dendrimers are suitable for the prolonged delivery of 5-fluorouracil [90]. Additionally, Gupta et al. employed PAMAM dendrimers to transport berberine, a drug with potential anti-cancer activity. They prepared two formulations: in the first, berberine was conjugated with the dendrimers, while in the second, the drug was encapsulated inside the PAMAM structure. They then went on to evaluate the biocompatibility and safety of these formulations in albino rats, observing that the conjugated form was safer than the encapsulated one in this animal model. Moreover, the half-life of berberine when delivered in the conjugated form was also significantly increased when compared to free delivery of the drug [91].

In another study, PPI dendrimers anchored with polysorbate 80 were used to deliver docetaxel in a brain tumor mouse model. These authors found that this nanosystem reduced the tumor volume and moreover, the median survival rate was almost twice as high as in the case of mice treated with PPI-DOX (without polysorbate 80), and more than double when compared to the free delivery of DOX [92]. Finally, when G5 L-lysine dendrimers were used as a vehicle for SN-38 (an active irinotecan metabolite) in a murine colorectal cancer xenograft model, sustained blood levels of SN-38 were detected, which led to significant tumor regression. Moreover, this conjugate exhibited reduced gastrointestinal toxicity compared to free delivery of the drug. They also observed that a regimen of 4 mg/kg SN-38 in 4 doses at weekly intervals extended the survival of the mice to 70 days after delivery of the final dose [93]. More in vivo studies on the use of dendrimers as carriers of anticancer drugs are described in Table 1.

The effect of dendrimer-based preparation has been also studied on animal models of ocular, metabolic, or inflammation diseases. Simvastatin was entrapped into amino-terminated, hydroxyl-terminated, and pegylated PAMAM dendrimers to compare its cholesterol-reducing effects with the free drug in male albino rats. Since entrapment increased simvastatin solubility, it was described that the drug residence time when used in this dendrimer formulation was 3–5 times longer than when the free drug was used. Additionally, drug absorption and elimination rates also decreased significantly, indicating the controlled release of simvastatin from these dendrimer formulations [101]. In a similar vein, phosphorus dendrimers were used to transport azabisphosphonate to target monocytes where they produced anti-inflammatory effects in a model of rheumatoid arthritis in both IL-1ra(−/−) mice (genetically modified mice with silenced gene encoding interleukin-1 receptor antagonist) and mice undergoing K/BxN serum transfer, an animal model of arthritis where the serum from arthritic transgenic K/BxN mice is transferred to naive mice and manifestations of arthritis occur a few days later. This nanosystem suppressed disease by reducing the levels of inflammatory cytokines. Moreover, inhibition of the colony-stimulating factor receptor promoted the maintenance of normal synovial membranes and cartilage, and prevented bone erosion and anti-osteoclastic activity, both in mouse and human cells [102]. In another study, PAMAM dendrimers were used as carriers for pilocarpine nitrate and tropicamide, resulting in prolonged drug residence time for the ophthalmic route when compared to free delivery of the drug [103]. Neutral high-generation phosphorus dendrimers bearing 48 (G3) or 96 (G4) bisphosphonate groups on their surface showed no toxicity and good solubility, as well as chemical stability in aqueous solutions. Furthermore, the anti-inflammatory activity of these neutral phosphorus dendrimers was high in a mouse model of sub-chronic inflammation [76]. Finally, in other works, amino-bis(methylene phosphonate)-capped phosphorus dendrimers prevented the development of experimental autoimmune encephalomyelitis and inhibited the progression of the established disease [104] (Table 2).

### 4.2. Dendrimers in Infectious Diseases

The antiviral activity of dendrimers is based on mimicking the anionic cell surface, and so dendrimers developed taking this approach also have a negative surface charge. Consequently, viruses bind to dendrimers not to cells, leading to a decrease in the viral infection rate. For example, PLL dendrimers with naphthyl residues were able to inhibit herpes simplex virus infection [112]; and moreover, PAMAM dendrimers modified with naphthyl sulfonate exhibited anti-HIV activity. In both cases, dendrimers inhibited virus entry into cells and replication. Furthermore, other PAMAM dendrimers combined with sialic acid have been studied as inhibitors of influenza A virus infection [113].

Excessive use of antibiotics has been one of the most important factors leading to the emergence of new antibiotic-resistant pathogen variants, meaning that research into new types of anti-bacterial agents remains of vital importance. In this context, dendrimers with a positive surface charge have shown antibacterial activity by binding to the anionic surface of bacterial membranes and damaging it, thereby causing lysis. For instance, PPI dendrimers with alkyl ammonium moieties have been studied to counteract anti-Gram-positive and anti-Gram-negative bacteria [114]. Furthermore, PLL dendrimers with mannosyl surface groups inhibited the adhesion of Escherichia coli to blood cells, making them good candidates as antibacterial drugs [115].

To date, only a few in vivo studies have been carried out to examine the activity of dendrimers against viruses and bacteria. In this sense, lysine dendrimers formulated with SPL7013 were used in mouse and guinea pig models to evaluate anti-herpes simplex virus activity, where they provided protection against infection at 10 mg/mL doses. However, further studies on guinea pigs showed that the optimal dose ranges from 30 to 50 mg/mL [116]. In another study, Landers et al. exploited a G4 PAMAM dendrimer carrying sialic acid to treat infection with three influenza variants in a mouse model. They showed that this nanosystem prevented infection by the H3N2 subtype but was unable to prevent pneumonitis caused by the other two virus types [113]. Elsewhere, researchers synthesized poly(phosphorhydrazone) dendrimers carrying mannose units with oligommanoside caps differing in size, number, and length. They showed that G3 dendrimers with 48 trimannoside caps reduced the neutrophil influx by targeting the DC-SIGN murine homolog, SIGN-related 1. Thus, this nanosystem showed great promise for the treatment of lung inflammation caused by Mycobacterium tuberculosis infection [117]. Even more interestingly, a G2 polyanionic carbosilane dendrimer, G2-S16, with a silica core and 16 sulfonate end-groups exerted anti-HIV-1 activity at an early stage of viral replication, blocking the viral protein gp120/CD4 interaction. Furthermore, topical vaginal administration of a 3% G2-S16 gel prevented HIV-1JR-CSF transmission by 84% in humanized (h)-BLT mice with no presence of HIV-1 RNA in vaginal lesions, thereby taking us one step forward the development of G2-S16-based vaginal microbicides to prevent vaginal HIV-1 transmission in humans [118].

### 4.3. Dendrimers in Gene Delivery

Gene therapy involves two basic actions: enhancing the expression of or silencing a specific gene, and depending on the application in question, different nucleic acids acting upon the cytosol and/or nucleus are used [119]. For silencing a specific gene, antisense oligonucleotides, or small-interfering RNA (siRNA) are generally used, reaching some of the later the clinical setting [120]. Viral vectors were initially employed for the delivery of genetic material [121,122]. However, because of their immunogenicity, carcinogenicity, and difficulties in large-scale production, their biocompatibility and efficacy has been challenged. Considering all the above, a limited number of approved and commercially used gene therapies are currently available [123]. Thus, the search for the ideal synthetic carrier for genetic material delivery is still underway. The main goals for any novel synthetic nanocarrier would be to efficiently deliver genetic material to patient cells without causing toxicity [124]. Of note, current in vitro studies indicate that dendrimers could be more efficient carriers for genetic materials than biological vectors [125]. Several in vivo studies have been conducted in the central nervous system (Table 3). For instance, PEGylated PAMAM dendrimers carrying angiopep-2, a ligand for lipoprotein receptor-related protein 1 that would facilitate BBB crossing, were used to target glioma cells and deliver tumor necrosis factor-related apoptosis-inducing ligand (TRAIL) as the therapeutic agent. This nanosystem exhibited good BBB penetration, and a favorable biodistribution and pharmacodynamic profile [126].

Elsewhere, cationic phosphorus dendrimers were used to deliver plasmid DNA encoding enhanced green fluorescent protein (GFP). Of note, this nanosystem did not show systemic toxicity after intratumoral injection of the formulation, suggesting that dendriplexes such as these could be good candidates for gene therapy [127]. Another study showed that compared to PAMAM dendrimers, polyethyleneimine (PEI) dendrimers transferred DNA to the airways in BALB/c mouse models more efficiently [128]. Similarly, Mekuria et al. used PAMAM dendrimers to create nanoclusters able to transfer DNA encoding GFP and p53. These authors proved that these dendriplexes were non-toxic to animals and also showed a high gene transfer rate [129]. In other work, poly(ether imine) (PETIM) dendrimers were also used to transport genetic material to enhance the immunogenicity and efficacy of a plasmid-based rabies vaccine in Swiss albino mice. The nanoformulations evaluated produced an earlier onset of a high-titer protective antibody response to rabies virus, thereby suggesting that PETIM dendriplexes were effective carriers for gene-based vaccines [130]. Moreover, Sheikh et al. utilized a polylysine-modified PEI (PEI-PLL) dendrimer to transport the vascular endothelial growth factor (VEGF) gene in a rat model of Parkinson disease where it prevented apoptosis and microglial activation [131].

**Table 3:** In vivo studies of dendrimers as carriers of genetic material. Transported active drugs are indicated in an italic font.

Dendrimer	Modification/ *Active Drug*	Outcome	Ref.
Phosphorus dendrimer	pyrrolidine ammonium	lack of systemic toxicity	[127]
Janus dendrimers	*mRNA*	higher transfection efficiency than the positive control organ specificity	[132]
	*siRNA*	ability to deliver Hsp27 siRNA to a castration-resistant prostate cancer mode gene silencing and potent anticancer activity	[133]
	*mRNA*	protonated ionizable amines play role in c hanging delivery from the lung to the spleen and/or liver replacing the interconnecting ester with the amide changed the delivery back to the lung	[134]
	*mRNA*	targeting the spleen, liver, and lymph nodes simplest synthetic vectors and the first system delivering equally to multiple organs	[135]
PAMAM	polyamidoamine	increased transfection in the lung more flexible structure after complexes activation	[136]
	fractured polyamidoamine	lower gene delivery efficiency in comparison with PEI dendrimers	[128]
	lipids	increased GFP expression improved serum biochemistry and hematological profile no tissue necrosis	[137]
	alkyl-carboxylate chain, PEG, and cholesteryl chloroformate	non-toxic, safe vector inhibition of tumor growth efficient gene delivery in TRAIL therapy	[138]
	4,4′-dithiodibutryic acid (DA)	lack of systemic toxicity efficient delivery of pDNA-p53 efficient cell cycle arrest at the G1 phase with upregulated p53 and p21 mRNA and protein expressions	[130]
	triethanolamine core	efficient delivery of siRNA gene silencing of Hsp27 significant anticancer activity in the prostate cancer	[139]
	hydroxyl-terminated	protecting of payload from degradation effective delivery of siRNA to the cells knockdown of GFP expression	[140]
	* plasmid DNA *	increased immunogenicity in comparison with the plasmid vector	[141]
Carbosilane dendrimer	* siRNA* * FITC *	detection of dendriplexes inside the brain efficient transport of siRNA into the brain	[142]
Phospholipid peptide dendrimers	-	more efficient intracellular uptake and endosome release better siRNA releasing ability more potent gene silencing and anticancer effects	[143]
Poly(ether imine)	*plasmid vaccine *	providing 100% protection against virus infection	[130]
PPI	*plasmid DNA *	induction of specific immunoglobulins and Th1 response	[144]
Modified dendrimer nanoparticle	*RNA *	efficient immunization	[145]
PEI-PLL	*VEGF gene *	prevented apoptosis and microglial activation in Parkinson’s disease beneficial effects of PEI-PLL-mediated VEGF gene delivery in the dopaminergic system	[131]

### 4.4. Dendrimers as Diagnostic Agents

The number of patient cases requiring diagnostic imaging is steadily increasing. Therefore, it will also be essential to develop specific and efficient methods to speed up diagnosis and enable timelier treatments to begin. The imaging agents currently used in magnetic resonance imaging, (MRI), computed tomography (CT), and scintigraphy are of low specificity, have a short half-life, and some toxicity [146], and so their combination with dendrimers is being explored to help overcome these limitations (Table 4). Indeed, dendrimers can increase the efficacy of imaging by, for example, increasing accumulation of the imaging agent in tumor lesions [51]. Moreover, by functionalizing the surface of nanoparticles, these nanosystems can be leveraged to target the specific proteins expressed in certain cell types [78]. Finally, in addition to improving the properties of imaging agents, dendrimers can act as drugs per se to support treatment [147]. Thus, all these approaches can be used to deliver imaging agents to target cells with more specificity, while also limiting the dose needed and, consequently, the side effects of the currently used imaging agents. For example, in one study, PAMAM dendrimers that target epidermal growth factor receptor-2 were used as a contrast agent for CT and MRI of HER-2-positive breast cancer. These authors showed that the use of this nanosystem significantly enhanced MRI signal intensity by about 20% and doubled the CT resolution and contrast in a mouse model, thereby suggesting that it can efficiently target and image HER-2-positive tumors [78].

PAMAM dendrimer-based gold nanoparticles have also been evaluated as a dual-modality contrast agent for MRI and CT imaging of breast cancer to efficiently provide imaging of a xenograft tumor model [148]. Moreover, a more complex nanosystem comprising PAMAM dendrimer-entrapped gold nanoparticles loaded with gadolinium chelator/Gd(III) complexes for targeted dual-mode MRI and CT imaging of small tumors and carrying a RGD peptide produced good imaging results in tumors overexpressing αvβ3 integrin [149]. Similarly, the use of amphiphilic Janus dendrimer-based dendrimersomes to transport an MRI contrast agent and prednisolone resulted in anti-tumor activity in an animal model of melanoma [150]. Finally, a multifunctional dendrimer entrapping gold nanoparticles and gadolinium, and also carrying FA to target xenografts was generated using the keratin-forming HeLa tumor cell line. When tested, this nanosystem showed good potential as an imaging agent, suggesting that such systems could be used to design imaging agents for the diagnosis of different cancer types [151].

**Table 4:** In vivo studies of dendrimers in diagnostics. Transported active drugs are indicated in an italic font.

Dendrimer	Modification/ *Active Drug*	Outcome	Ref.
PAMAM	encapsulated gold nanoparticles, chelated gadolinium, and *anti-human HER-2 antibody*	enhancing of MRI signal intensity by approx. 20% improving CT resolution two times	[78]
	thiolated cyclopeptide-based gold nanoparticle *entrapped gold nanoparticles loaded with gadolinium chelator/Gd(III) complexes*	usability as a dual-mode nanoprobe for targeted CT/MR imaging of different types of αvβ3 integrin-overexpressing cancer	[149]
	poly(amidoamine)/gold nanoparticles *gadolinium chelate*	biocompatibility efficient cellular uptake usability in dual-mode MR/CT imaging of the xenograft tumor model after intravenous injection of the particles	[148]
	FA-modified *entrapped gold nanoparticles loaded with gadolinium*	high intensity of radiation suppression improved MRI contrast usability in dual mode nanoprobes for targeted CT/MR imaging xenograft tumor model in vivo via the FA receptor-mediated active targeting pathway	[149]
	*gadolinium*-*loaded dendrimer-entrapped gold nanoparticles*	extended blood circulation time total clearance within 24 h usability in dual-mode CT/MR imaging of the heart, liver, kidney, and bladder	[152]
	surface-PEGylated Gd-PAMAM dendrimers	higher relaxivities stability in the blood decreased plasma clearance usability as a carrier for diagnostic or theranostic agents	[153]
	N-succinimidyl (S)-acetyl(thiotetraethylene glycol with *Gadolinium*	possessed a circulation half-life of >1.6 h significant contrast enhancement in the abdominal aorta and kidneys for as long as 4 h reduced circulation time as a result of thiol–disulfide exchange, and the degradation products were rapidly excreted via renal filtration	[154]
	Arg-Gly-Asp (RGD)-modified conjugated with *Fe_3_O_4_ NPs*	high affinity for C6 cells that overexpress α_v_β_3_ receptors in mice excellent potential for use as contrast agents for targeted T_2_ MR imaging of specific tumors.	[155]
Janus dendrimers	*GdDOTAGA(C18)_2_ and prednisolone phosphate*	contrast enhancement in the tumor area good efficiency and displayed anti-tumor activity usability in theranostic applications	[150]
	*GdDOTAGA(C18)_2_*	improved relaxivity long-term in vivo retention of the nanoprobe	[156]
poly(propylene imine) dendrimers	densely organized maltose shell (MAL DS) *tetraazacyclododecane tetraacetic acid (DOTA) ligands*	fast metabolization and total clearance in 48 h efficient contrast agent for MR imaging aorta, renal artery, kidney, and bladder	[157]
peptide dendrimers	*gadolinium*	higher signal intensity enhancement much higher Gd(III) concentration in blood usability as an MRI probe	[158]
triazine dendrimer	*gadolinium*	high in vivo r1 relaxivity, desirable pharmacokinetics, and well-defined structure	[159]
folic acid- and gadolinium-labeled dendrimer	FA/graphene oxide *gadolinium*	long blood circulation time excellent magnetic resonance angiography (MRA) images with high-resolution vascular structures	[160]
polyester dendrimer	zwitterionized	minimal long-term Gd^3+^ retention in all organs and tissues degraded into small fragments significant capability of enhancing the MRI of metastases in the liver	[161]

## 5. Future Perspectives

The continuous development of nanotechnology makes it possible to produce increasingly sophisticated and complex dendrimers that have a wide spectrum of applications in medicine. Moreover, chemical modifications of their surfaces can further expand their capabilities as nanocarriers. Additionally, some dendrimers can act as drugs in and of themselves, as indicated in Section 4.1, thereby also contributing to treatment effectivity. Of note, further work will be essential to thoroughly understand the biological properties and actions of dendrimers on living organisms. To date, several in vitro studies in which dendrimers have proven to be excellent carriers have already been published. Unfortunately, not all the results from this initial research could be confirmed in in vivo preclinical phase drug development trials focusing on selecting optimal drugs and dosages, or in pharmacokinetic and pharmacodynamic studies. Thus, to date, there is scarce information about the chemical stability and long-term toxicity of these nanosystems, and standardized methods to evaluate nanoparticles are also scarce.

Therefore, so far, only a few dendrimer-based formulations have reached clinical trials, including safety and pharmacokinetic studies for hydroxyl-polyamidoamine dendrimer-N-actylcysteine conjugates (NCT03500627), hydroxyl-terminated PAMAM (NCT05105607), or as delivery agents for therapeutic compounds to treat COVID disease (NCT05208996), OP-101, a hydroxyl-polyamidoamine dendrimer-N-acetylcysteine conjugate [162], Phase I/II poly-L-lysine dendrimers for advanced non-Hodgkin lymphoma alone (NCT04214093) or in combination with voriconazole (NCT05205161), and poly-L-Lysine conjugated to a complex of 188Rhenium–ligand (nitro-imidazole-methyl-1,2,3-triazol-methyl-di-[2-pycolyl] amine) for inoperable liver tumor resistant to other therapies (NCT03255343). Although some compounds have successfully moved from in vivo studies to clinical trials, to date, only one dendrimer-based formulation has passed clinical trials to reach the market: VivaGel^®^ (SPL7013), a G4 polylysine dendrimer that was approved for the treatment of bacterial vaginosis and protection against HIV [10]. However, the large amount of research data already published on dendrimers indicates that this fascinating family of nanoparticles has wide-ranging potential in the pharmaceutical industry, especially for applications in drug delivery systems. Indeed, the use of cutting-edge technologies has made it possible to develop advanced systems for delivering active agents into living systems. However, further research on dendrimers will still be needed to allow for medicine to reach previously unknown levels of sophistication through nanosystems, thereby making it possible to manage diseases that currently lack effective treatments.

## Figures and Tables

**Table 1:** In vivo studies of dendrimers as carriers for anti-cancer drugs. Transported active drugs are indicated in an italic font.

Dendrimer	Modification/ *Active Drug*	Outcome	Ref.
PAMAM	*cisplatin*	selective accumulation in the tumor decreased toxicity in comparison with free cisplatin	[94]
	PEG and *5 fluorouracil*	lack of significant hematological disturbances	[90]
	β-cyclodextrin and oligoarginine peptide entrapped gold nanoparticles and *dexamethasone*	improved hearing in C57/BL6 mice more effective tympanic injection than posterior ear injection, muscle injection, and tail vein injection	[95]
	L-cysteine and *doxorubicin*	L-cysteine acts as a radiosensitizer inhibition of cancer growth	[89]
	*berberine*	longer half-life and AUC	[91]
	folic acid and borneol	tumor growth inhibition median survival time prolonged compared to free doxorubicin	[96]
	cholesteryl chloroformate and alkyl-PEG	significantly decreased tumor growth rate	[97]
	dendrimer–gold hybrid structure	effective anti-tumor agent accurate CT imaging usability in combined detection of therapy of different adenocarcinomas via an active, MUC-1-mediated targeting pathway	[98]
PPI	Polysorbate 80	anti-cancer activity in brain tumor longer survival time of the case of DTX–P80-PPI than DTX–PPI higher targeting efficiency and biodistribution of ligand-conjugated dendrimer into the brain	[92]
phosphorus dendrimers	*doxorubicin* *(DOX)*	good intrinsic anticancer activity collective action with DOX to take down breast cancer via the upregulation of Bax, PTEN, and p53 proteins for enhanced cell apoptosis	[87]
Janus dendrimers	*doxorubicin (DOX)*	minimal systemic toxicity half-life approx. 16 h 9 times higher tumor uptake in comparison with free DOX complete tumor regression 100% survival of the mice antitumor effect of dendrimer–DOX similar to liposomal DOX	[99]
	*cytarabine and daunorubicin*	superior therapeutic activity compared to free drug cocktails prolonged maintenance of synergistic drug ratios in the bone marrow	[100]
peptide dendrimers	*doxorubicin*	increased doxorubicin concentration in the tumor enhanced anticancer efficacy of doxorubicin and gemcitabine	[88]
polylysine dendrimers	*polyoxazoline*	prolonged circulation of the dendrimer survival period of the mice extended beyond 70 days following the final dose	[93]
rotaxane dendrimer	*chlorambucil*	accumulation in the reticuloendothelial system enriching spleen and liver	[16]

**Table 2:** In vivo studies of dendrimers as carriers for drugs in non-cancer diseases. Transported active drugs are indicated in an italic font.

Dendrimer	Modification/ *Active Drug*	Outcome	Ref.
Phosphorus dendrimer	azabisphosphonate (ABP)	efficient anti-inflammatory activity	[76]
	azabisphosphonate (ABP)	anti-inflammatory activity selective targeting to monocytes anti-osteoclastic activity	[102]
	amino-bis(methylene phosphonate)	preventing of autoimmune encephalomyelitis development inhibition of established disease progression redirection of pathogenic myelin-specific CD4+ T cell response toward IL-10 production	[104]
	amino-bis(methylene phosphonate)	inhibition of the onset and development of experimental arthritis lack of adverse response lack of lesion or non-physiological occurrence	[105]
	*carteolol*	no eye irritation 2.5 times larger quantity of mix of carteolol and dendrimer in comparison with free carteolol	[106]
Janus dendrimers	carboxybetain and α-lactalbumin	prolonged pharmacokinetic profiles of payloads in comparison with the PEGylated nanocarriers	[107]
PAMAM	amino-terminated	improved diabetes-induced vascular remodeling and dysfunction inhibited EGFR-ERK1/2-ROCK signaling	[108]
	amino-terminated	intraperitoneal and subcutaneous administration are the most effective in suppressing the long-term markers of hyperglycemia the lowest incidence of side effects was observed after subcutaneous administration subcutaneous injection is the best way to compromise moderate PAMAM toxicity and effective reduction in the markers of long-term severe hyperglycemia	[109]
		reduced levels of blood glucose glycated hemoglobin or protein oxidation, cholesterol, and triglycerides higher terminal blood glucose in PAMAM-treated animals	
	amino-terminated	reduction of blood glucose concentration and hallmarks of late diabetic complications reduced diabetes-induced permeabilization of BBB enrichment of the biochemical hallmarks of severe hyperglycemia.	[110]
	*simvastatin*	reduction in the increase in cholesterol level approx. 2 times and in triglyceride and low-density lipoprotein levels better pharmacokinetic performance in comparison with free drug three-to-five times higher residence time in comparison with free drug controlled release of simvastatin decreasing absorption and elimination rates	[101]
Lysine dendrimers	mannose	accelerated diabetes wound repair after topical administration increased closure rate, collagen deposition, and angiogenesis, production of TGF-β1	[111]

## Data Availability

No new data were generated for this manuscript.
